# Prognostic value of nonangiogenic and angiogenic growth patterns in non-small-cell lung cancer

**DOI:** 10.1038/sj.bjc.6602134

**Published:** 2004-08-24

**Authors:** P Sardari Nia, C Colpaert, B Blyweert, B Kui, P Vermeulen, M Ferguson, J Hendriks, J Weyler, F Pezzella, E Van Marck, P Van Schil

**Affiliations:** 1 Department of Thoracic and Vascular Surgery, University Hospital of Antwerp, Belgium; 2 Translational Cancer Research Group, Oncology Centre, General Hospital Sint-Augustinus and University of Antwerp, Antwerp, Belgium; 3 Department of Pathology, University Hospital of Antwerp, Belgium; 4 Cancer Research UK Tumour Pathology Group, Nuffield Department of Clinical Laboratory Sciences, John Radcliffe Hospital, Oxford, UK; 5 Department of Epidemiology and Social Medicine, University Antwerp, Belgium

**Keywords:** non-small-cell lung cancer, growth pattern, prognosis, angiogenesis, nonangiogenic growth, survival analysis

## Abstract

An essential prerequisite of nonangiogenic growth appears to be the ability of the tumour to preserve the parenchymal structures of the host tissue. This morphological feature is visible on a routine tissue section. Based on this feature, we classified haematoxylin and eosin-stained tissue sections from 279 patients with non-small-cell lung cancer into three growth patterns: destructive (angiogenic; *n*=196), papillary (intermediate; *n*=38) and alveolar (nonangiogenic; *n*=45). A Cox multiple regression model was used to test the prognostic value of growth patterns together with other relevant clinicopathological factors. For overall survival, growth pattern (*P*=0.007), N-status (*P*=0.001), age (*P*=0.020) and type of operation (*P*=0.056) were independent prognostic factors. For disease-free survival, only growth pattern (*P*=0.007) and N-status (*P*<0.001) had an independent prognostic value. Alveolar (hazard ratio=1.825, 95% confidence interval=1.117–2.980, *P*=0.016) and papillary (hazard ratio=1.977, 95% confidence interval=1.169–3.345, *P*=0.011) growth patterns were independent predictors of poor prognosis. The proposed classification has an independent prognostic value for overall survival as well as for disease-free survival, providing a possible explanation for survival differences of patients in the same disease stage.

Although a large number of papers have been published evaluating prognostic factors in cancer (Cannistra, 2000), TNM-staging is still the most important tool used to estimate prognosis for lung cancer patients and to select the best possible combination of treatment modalities. Although TNM-staging gives an accurate estimate of the progression of the disease at the time of diagnosis, it does not always account for survival differences. Resected stage I non-small-cell lung cancer (NSCLC) is a typical example with wide differences in survival for tumours resected at an identical early stage.

The last few decades have been characterised by a move in cancer research from a reductionistic approach focusing mainly on the cancer cell itself to a greater appreciation of the importance of interactions between cancer cells and stroma. This has resulted in much new information concerning molecular–biological processes that take place in the stroma, one of which is the formation of new blood vessels, angiogenesis.

In the past 10 years, more than 100 papers have been published studying various components of angiogenesis and their relation to prognosis in lung carcinoma, but results so far are inconclusive or awaiting confirmation and clinical practice has not been altered (Cox *et al*, 2000; Shepherd and Sridhar, 2003; Yano *et al*, 2003).

The hypothesis that tumour growth is dependent on angiogenesis (Folkman, 1990), which prevailed for many years, has been challenged with the description of nonangiogenic growth. In a study of 500 NSCLC, an ‘alveolar’ growth pattern was described, in which the tumour cell nests filled the alveolar spaces without destruction of lung parenchyma, co-opting the septal blood vessels (Pezzella *et al*, 1997). A subsequent study confirmed this growth pattern to be nonangiogenic (Passalidou *et al*, 2002). Nonangiogenic growth patterns have also been described in liver (Vermeulen *et al*, 2001; Stessels *et al*, 2004), lymph node (Vermeulen *et al*, 2002b) and lung metastases (Pezzella, 2000).

An essential prerequisite for nonangiogenic growth appears to be the ability of the tumour to preserve the stromal architecture of the host tissue. With the advent of antiangiogenic drugs, the vasculo-morphological growth pattern may have a clinical value as it could be used as a surrogate marker of angiogenesis. Additionally, these growth patterns may have prognostic importance. The prognostic value of the alveolar, nonangiogenic growth pattern has been suggested in four studies (Pastorino *et al*, 1997; Pezzella *et al*, 1997; Offersen *et al*, 2001; Funai *et al*, 2003). The aim of this study was to test the prognostic value of growth patterns using a modification of the classification of NSCLC into growth patterns previously proposed by Pezzella *et al* (1997).

## PATIENTS AND METHODS

### Study population

All relevant clinical information was gathered retrospectively from 369 consecutive patients who underwent curative surgical resection for primary NSCLC at the University Hospital of Antwerp between January 1991 and January 2001. Standardised forms were completed based on the in-patient medical records and the medical records of the consultant respiratory physician in our department or in the referral hospital. All patients were operated on by the same surgeon (PVS). The pathological TNM-staging of NSCLC was used based on histopathology reports and on the perioperative findings (Mountain, 1997). The disease-free interval was defined as disease-free survival time or the interval between surgery and relapse. Relapse was defined as local recurrence, metastasis or both. Recurrence was confirmed on both clinical, radiological and/or biopsy results. A total of 90 patients were excluded from further analyses: 26 (7.0%) due to perioperative mortality (two died during the operation and 24 within 30 days or during the same hospitalisation), 26 who had received induction chemotherapy, 11 patients with metastases observed at the time of surgery, six patients with a double tumour at the time of diagnosis, five patients who underwent a double surgical procedure (coronary bypass and lobectomy), three patients who had had metastasectomy for a solitary brain metastasis prior to lung operation and 13 due to poor quality tissue sections or tissue sections without recognisable interface between tumour and normal lung tissue. A total of 279 patients (241 men and 38 women) were included in the study.

### Morphological analysis

**Figure 1:**
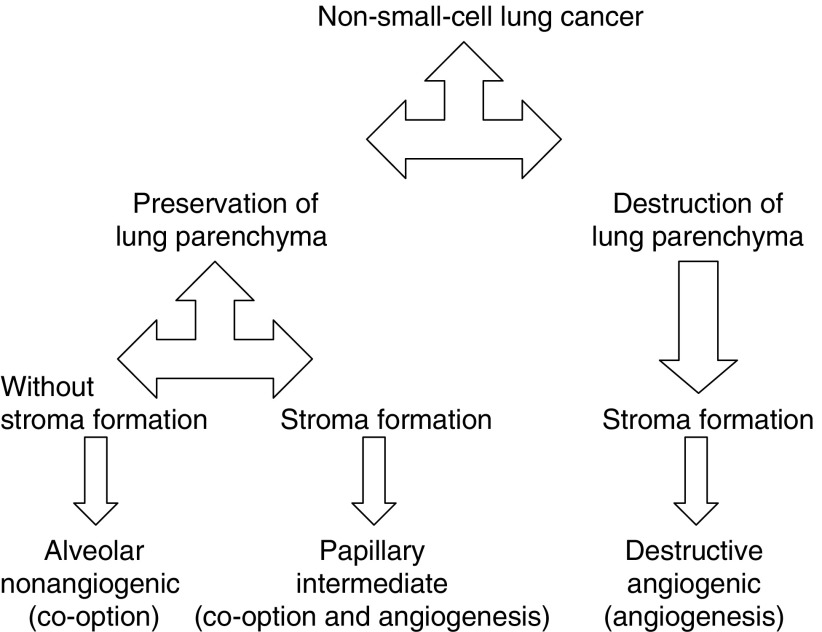
Classification of non-small-cell lung cancer.

Haematoxylin and eosin (H&E)-stained tissue sections from all tumour blocks from each patient were examined. Tumours were classified according to growth patterns based on two considerations: firstly, preservation or destruction of lung parenchyma at the lung–tumour interface and, secondly, presence or absence of a tumour-associated stroma at the lung–tumour interface (Figure 1

). The interface was defined as a field of × 100 magnification at the edge of the tumour, containing only tumour tissue, next to normal lung tissue. Based on these criteria, tumours were classified into three growth patterns as follows:

1. Destructive growth pattern (angiogenic growth pattern): destruction of lung parenchyma with the presence of tumour-associated stroma at the interface.
2. Papillary growth pattern (intermediate growth pattern): preservation of the alveolar structure of the lung parenchyma at the interface with formation of stromal stalks containing capillary vessels originating from the alveolar septa, suggesting co-option of alveolar blood vessels with subsequent angiogenesis.
3. Alveolar growth pattern (nonangiogenic): preservation of the alveolar structure of lung parenchyma with co-option of septal blood vessels and without evidence of new stroma formation at the interface. In this growth pattern, solid tumour cell nests fill the alveolar spaces, often with the presence of necrosis in the centre of these nests. This group does not include bronchiolo-alveolar carcinoma (BAC), which is characterised by orderly replacement of pneumocytes by tumour cells along the alveolar septa without infiltration, necrosis or fibrovascular proliferation. The studied patient population contained only three BACs according to WHO classification. However, the patients presenting these tumours had different nodules in different lung lobes at the time of diagnosis. So these patients were excluded, because they had M1 status at the time of operation.

The growth patterns described are characteristics of the whole tumour tissue at the interface. For a tumour to be classified as alveolar or papillary, this tumour growth had to be present throughout the whole interface. In very few cases, a mixed growth pattern was observed at the interface. In these few cases, a major destructive component was always observed with a minor alveolar or papillary component. So, these tumours were included in the destructive group.

The growth patterns described are morphological features different from the histiotypes and are based on biological behaviour of the tumour tissue with respect to the lung parenchyma; alveolar growth pattern must not be confused with bronchiolo-alveolar histological subtype of adenocarcinomas.

The proposed classification is based upon the study of Pezzella *et al* as shown in Table 1

**Table 1:** Modified classification of NSCLC based on growth patterns

**Growth pattern**	**Determination**	**Vascularisation at interface**	**Destruction of lung parenchyma at interface**	**Formation of stroma at interface**	**Corresponding growth pattern according to Pezzella *et al* (1997)**
Destructive (angiogenic)	At the interface	Only newly formed vessels	Destruction	Present	Diffuse and basal
Papillary (intermediate)	At the interface	Newly formed vessels admixed with pre-existing vessels	No destruction	Present	Papillary
Alveolar (nonangiogenic)	At the interface	Pre-existing vessels	No destruction	Absent	Alveolar (entirely alveolar)+partially alveolar (basal, diffuse)

. The following modifications were made: (1) the ‘diffuse’ and ‘basal’ categories were merged into the ‘destructive’ group; (2) growth patterns were based only on the properties of the interface; (3) classification was made using H&E-stained tissue sections without additional sections immunostained for vascular markers.

### Statistical analysis

Survival effects were evaluated using Kaplan–Meier plots, and the differences were assessed by the log-rank test. In order to assess the presence of confounding, the distribution of known prognostic factors between growth patterns was analysed by the *χ*^2^ test for categorical variables. Continuous variables, age and size of the tumour were tested for normality with the Kolmogorov–Smirnov test. The distribution of these factors between growth patterns was assessed by the Kruskal–Wallis test, because these factors were not normally distributed.

Potential confounders were entered into a Cox multiple regression model for overall survival as well as for disease-free survival. The model building was guided by the influence of the inclusion of these factors on the regression coefficient. The disease-free survival analysis was performed for 267 patients because in 12 cases the disease-free survival time could not be exactly determined. The estimates were presented with a 95% confidence interval or with standard error. The analyses were performed with SPSS for Windows (Release 11.5 SPSS Inc.).

## RESULTS

The median patient age at the time of the operation was 66 years (range: 39–87). According to postoperative TNM-staging, 77 (27.6%) patients were in stage IA, 90 (32.3%) patients in stage IB, 12 (4.3%) patients in stage IIA, 60 (21.5%) patients in stage IIB, 17 (6.1%) patients in stage IIIA and 23 (8.2%) patients were in stage IIIB. A total of 52 (18.6%) patients received postoperative radiotherapy. The median follow-up of the whole group was 34 months (range: 2–135). Kaplan–Meier survival estimate at 5 years for the whole group was 54.7% (standard error (s.e.)=3.5%) and estimates for stage IA and IB were 66.4% (s.e.=6.4%) and 63.3% (s.e.=5.9%), respectively.

At the moment of final evaluation for this study (January 2003), 163 (58.4%) patients were alive of whom 137 (49.1%) were disease free, 119 (42.7%) had relapsed, 16 (5.7%) had developed a second primary and 116 (41.6%) had died (Table 2

**Table 2:** Status at the end of follow-up

	**Alive**	**Dead**	**Total**
Recurrence	25	94	119
Second primary	10^a^	6^a^	16^a^
Dead of other causes	—	13^a^	13^a^
Not known	1	3	4
Disease free	137	19	156
Total	163	116	279

a Patients with a second primary and dead of other causes were considered disease free.

). In total, 31% of the patients with a relapse had developed local recurrence, 67% had developed metastases and 2% had developed both.

**Figure 2:**
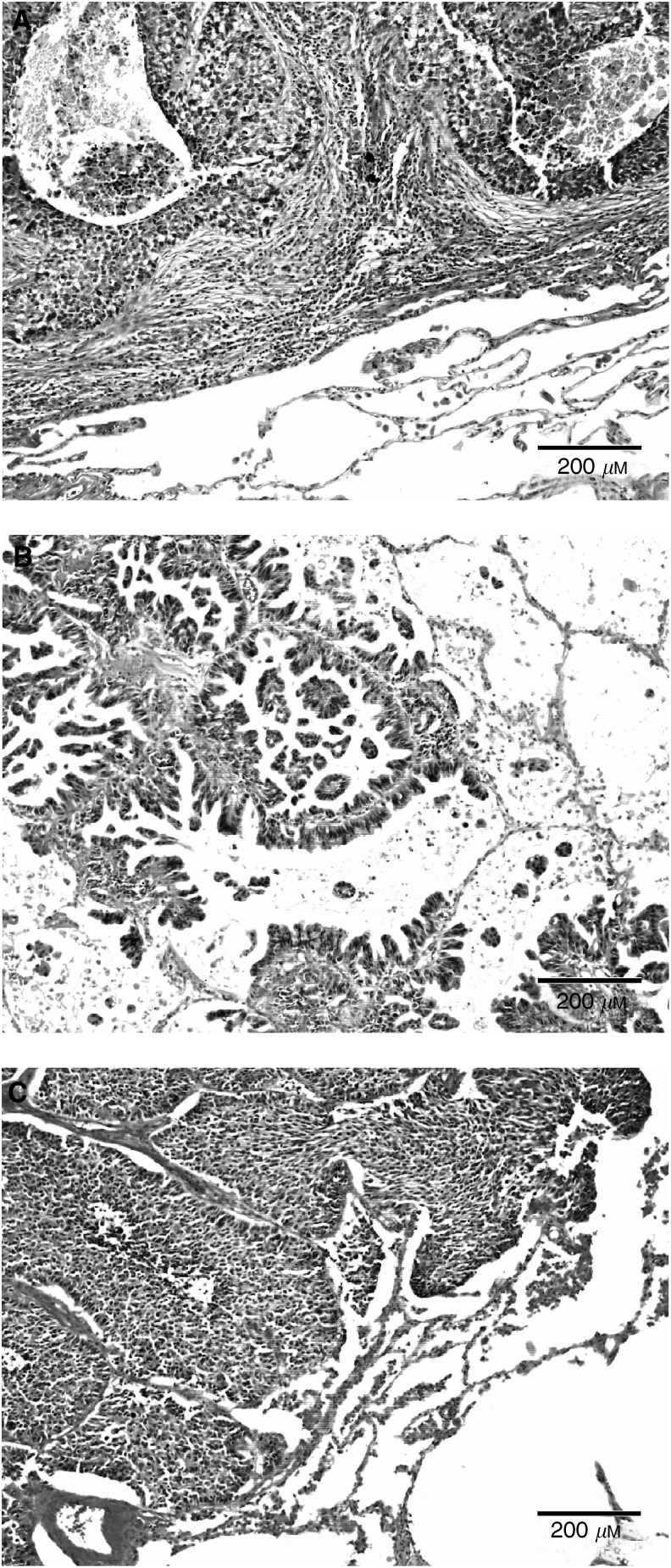
Growth patterns of non-small cell lung cancer are visible on routine H&E-stained tissue sections. (**A**) Destructive growth pattern (angiogenic): parenchymal structures of the lung are not preserved, but replaced by carcinoma cells and tumour-associated stroma. (**B**) Papillary growth pattern (intermediate): the lung parenchyma is preserved with formation of fibrovascular stalks originating from the alveolar septa. (**C**) Alveolar growth pattern (nonangiogenic): the tumour cell nests fill the alveolar spaces with preservation of alveolar septa and without formation of a tumour-associated stroma.

Based upon our classification, 196 (70.3%) patients had a tumour with a destructive growth pattern (Figure 2A

), 38 (13.4%) patients had a tumour with a papillary growth pattern (Figure 2B) and 45 (16.1%) patients had a tumour with an alveolar growth pattern (Figure 2C).

The Kaplan–Meier survival analyses of different clinicopathological factors for overall survival and disease-free survival are presented in Table 3

**Table 3:** Univariate analysis of different clinicopathological factors for overall survival (OS) and disease-free survival (DFS)

		**OS (months)**			**DFS (months)**		
	***n***	**Median (s.e.^b^)**	**95% CI^a^**	***P*-value**	**Median (s.e.)**	**95% CI**	***P*-value**
*Gender*							
Male	241	77 (13)	51–103		Nr	—	
female	38	Nr^c^	—	0.2677	84 (37)	12–156	0.8626
							
*Histiotype*							
Adenocarcinoma	93	59 (10)	40–78		Nr	—	
Squamous	128	87 (11)	66–108	0.6150	Nr	—	0.4168
Large cell	42	Nr	—	0.8902	Nr	—	0.8088
other	16	Nr	—	0.7518	71 (44)	0–156	0.9604
							
*Localisation*							
Left	122	69 (12)	46–92		84 (30)	26–124	
right	157	90 (11)	65–111	0.1260	Nr	—	0.1295
							
*Type of operation*							
(Bi)lobectomy	192	94 (12)	70–118		Nr	—	
Pneumonectomy	87	35 (7)	21–49	0.0004	22 (6)	10–34	0.0005
							
*T-status*							
T1	89	94 (15)	64–124		102 (17)	68–136	
T2	147	72 (15)	44–100	0.1050	Nr	—	0.0646
T3	20	Nr	—	0.8880	Nr	—	0.2792
T4	23	37 (9)	19–55	0.0151	18 (2)	15–21	0.0011
							
*N-status*							
N0	198	102 (10)	83–121		Nr	—	
N1	64	39 (8)	23–55	0.0002	27 (12)	3–51	0.0009
N2	17	29 (8)	13–45	0.0001	12 (3)	5–19	<0.0001
							
*Stage*							
St IA	77	94 (15)	65–123		Nr	—	
St IB	90	90 (10)	70–110	0.6234	Nr	—	0.3330
St IIA	12	46 (2)	41–51	0.0623	22 (21)	0–63	0.0135
St IIB	60	58 (24)	12–104	0.0098	71 (28)	17–125	0.0055
St IIIA	17	29 (7)	14–44	0.0001	12 (7)	0–25	<0.0001
St IIIB	23	37 (9)	19–55	0.0045	18 (2)	15–21	0.0001
							
*Radiotherapy*							
No	227	90 (11)	68–112		Nr	—	
received	52	33 (10)	14–52	0.0074	21 (4)	14–28	0.0003
							
*Growth pattern*							
Destructive	196	96 (13)	71–121		Nr	—	
Papillary	38	48 (10)	28–68	0.1640	Nr	—	0.3039
Alveolar	45	59 (25)	10–108	0.0130	22 (11)	0–44	0.0070

a 95% CI=95% confidence interval.

b s.e.=standard error.

c Not reached.

**Figure 3:**
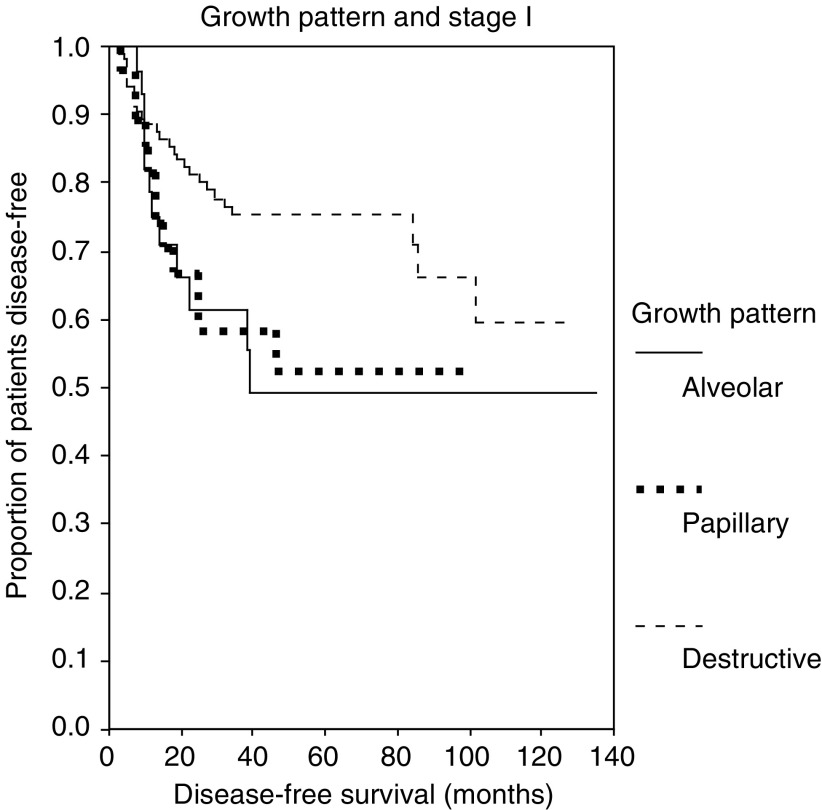
Disease-free survival according to growth pattern for patients in stage I (destructive *vs* papillary growth, *P*=0.0455 and destructive *vs* alveolar growth pattern, *P*=0.0465).

. Factors that were significant for overall survival and disease-free survival were the type of operation, the T-status, the N-status, the stage of the disease, postoperative radiotherapy and growth pattern. Kaplan–Meier survival estimates at 5 years for destructive, papillary and alveolar growth pattern were 60.7% (s.e.=4.0%), 38.9% (s.e.=9.6%) and 43.0% (s.e.=10%), respectively. Kaplan–Meier survival curves for disease-free survival of patients in stage l according to growth pattern are shown in Figure 3

.

The association of different clinicopathological findings with growth patterns is presented in Table 4

**Table 4:** Association of different clinicopathological factors with growth patterns

**Items**	**Destructive *N* (%)**	**Papillary *N* (%)**	**Alveolar *N* (%)**	***P*-value**
*Number of cases*	196 (70.3)	38 (13.4)	45 (16.1)	
				
*Gender*				0.069
Female	21 (10.7)	9 (23.7)	8 (17.8)	
Male	175 (89.3)	29 (76.3)	37 (82.2)	
				
*Age*^a^ *(years)*	65 (40–87)	65 (42–77)	66 (39–80)	0.324
				
*Histiotype*				<0.0001
Adenocarcinoma	43 (21.9)	38 (100)	12 (26.7)	
Squamous cell	110 (56.1)	—	18 (40.0)	
Large cell	29 (14.8)	—	13 (28.9)	
others	14 (7.1)	—	2 (4.4)	
				
*Localisation*				0.264
Left lung	89 (45.4)	12 (31.6)	21 (46.7)	
Right lung	107 (54.6)	26 (68.4)	24 (53.3)	
				
*Stage*				0.245
IA	47 (24.0)	16 (42.1)	14 (31.1)	
IB	60 (30.6)	13 (34.2)	17 (37.8)	
IIA	8 (4.1)	2 (5.3)	2 (4.4)	
IIB	51 (26.0)	3 (7.9)	6 (13.3)	
IIIA	14 (7.1)	1 (2.6)	2 (4.4)	
IIIB	16 (8.2)	3 (7.9)	4 (8.9)	
				
*T-status*				0.194
T1	55 (28.1)	18 (47.4)	16 (35.6)	
T2	107 (54.6)	16 (42.1)	24 (53.3)	
T3	18 (9.2)	1 (2.6)	1 (2.2)	
T4	16 (8.2)	3 (7.9)	4 (8.9)	
				
*N-status*				0.282
N0	132 (67.3)	32 (84.2)	34 (75.6)	
N1	50 (25.5)	5 (13.2)	9 (20.0)	
N2	14 (7.1)	1 (2.6)	2 (4.4)	
				
*Type of operation*				0.081
(Bi)lobectomy	129 (65.8)	32 (84.2)	31 (68.9)	
Pneumonectomy	67 (34.2)	6 (15.8)	14 (31.1)	
				
*Size of the tumor*^a^ *(cm)*	4.00 (1–16)	2.95 (1–13)	3.10 (1–14)	0.049
				
*Radiotherapy*				0.385
No	157 (80.1)	34 (89.5)	36 (80.0)	
Received	39 (19.9)	4 (10.5)	9 (20.0)	

a For continuous variables, figures are median (range).

. There were no significant differences in distribution of clinicopathological factors between different growth patterns, except for histiotype and size of the tumour. The papillary growth pattern was exclusively seen in adenocarcinomas (in 100%) and tumours with this growth pattern tended to be smaller.

### Cox multiple regression analyses

Based on the results obtained by Kaplan–Meier survival analyses and descriptive analyses, the following factors were entered into Cox multiple regression analyses: age (as continuous variable), gender (male or female), type of operation ((bi)lobectomy or pneumonectomy), histiotype (adenocarcinoma, squamous cell carcinoma, large-cell carcinoma or others), postoperative radiotherapy or not, localisation (left or right lung), N-status (N0, N1 or N2), T-status (T1, T2, T3 or T4), growth pattern (destructive, papillary or alveolar) and the interaction variable (growth pattern × histiotype). This interaction variable was included because a strong association was found between histiotype and growth pattern in descriptive analyses (Table 4).

For overall survival, alveolar growth pattern, papillary growth pattern, advanced N-status, higher age and pneumonectomy were independent predictors of poor prognosis (Table 5

**Table 5:** Cox multiple regression for overall survival

**Factor**	**Hazard ratio**	**95% CI^a^**	***P*-value**
*Growth pattern*			0.007
Destructive	1		
Papillary	1.977	1.169–3.345	0.011
Alveolar	1.825	1.117–2.980	0.016
			
*N-status*			0.001
N0	1		
N1	1.969	1.244–3.118	0.035
N2	2.961	1.526–5.748	0.009
			
*Age*	1.029	1.005–1.054	0.020
			
*Type of operation*			
(bi)lobectomy	1		
Pneumonectomy	1.530	0.989–2.369	0.056

a 95% CI=95% confidence interval.

). The interaction variable was not significant (*P*=0.574).

For disease-free survival, alveolar growth pattern, papillary growth pattern and advanced N-status were independent predictors of local recurrence and/or metastasis (Table 6

**Table 6:** Cox multiple regression for disease-free survival

**Factor**	**Hazard ratio**	**95% CI^a^**	***P*-value**
*Growth pattern*			0.007
Destructive	1		
Papillary	1.665	0.966–2.871	0.067
Alveolar	2.023	1.264–3.235	0.003
			
*N-status*			<0.001
N0	1		
N1	1.795	1.277–3.062	0.002
N2	2.717	1.930–5.060	<0.001
			
*Type of operation*			
Lobectomy	1		
Pneumonectomy	1.478	0.946–2.309	0.089

a 95% CI=95% confidence interval.

). The interaction variable was not significant (*P*=0.427).

### Cox multiple regression analyses for stage IA and IB

For clinical relevance, a subanalysis was performed for patients in stage IA and IB and the following factors were entered into Cox multiple regression analyses: age (as continuous variable), gender (male or female), type of operation ((bi)lobectomy or pneumonectomy), histiotype (adenocarcinoma, squamous cell carcinoma, large-cell carcinoma or others), localisation (left or right lung), T-status (T1 or T2), growth pattern (destructive, papillary or alveolar) and the interaction variable (growth pattern × histiotype).

For overall survival, alveolar growth pattern, papillary growth pattern and higher age were independent predictors of poor prognosis (Table 7

**Table 7:** Cox multiple regression for overall survival for patients in stage I

**Factor**	**Hazard ratio**	**95% CI^a^**	***P*-value**
*Growth pattern*			0.023
Destructive	1		
Papillary	2.262	1.183–4.325	0.014
Alveolar	1.937	0.980–3.825	0.057
Age	1.050	1.012–1090	0.010

a 95% CI=95% confidence interval.

). The interaction variable was not significant (*P*=0.689).

For disease-free survival, alveolar and papillary growth pattern were the only independent predictors of local recurrence and/or metastasis (Table 8

**Table 8:** Cox multiple regression for disease-free survival for patients in stage I

**Factor**	**Hazard ratio**	**95% CI^a^**	***P*-value**
*Growth pattern*			0.056
Destructive	1		
Papillary	1.959	0.988–3.882	0.054
Alveolar	1.989	1.004–3.942	0.049

a 95% CI=95% confidence interval.

). The interaction variable was not significant (*P*=0.412).

## DISCUSSION

We have found that growth pattern is an independent prognostic factor in patients with NSCLC. Interestingly, growth pattern is one of the most important independent prognostic factors in stage I NSCLC, giving a possible explanation for survival differences among patients with stage I disease. The clinical relevance of this finding is important since patients at risk for early relapse and early mortality can be recognised based on a reproducible characteristic of the tumour tissue, the growth pattern, visible on a routine H&E-stained tissue section.

The classification of NSCLC into growth patterns was first proposed by Pezzella *et al* (1997). Based upon frequency tables, they observed that the patients with a purely alveolar growth pattern (alveolar growth throughout the whole tumour section) had a higher recurrence rate than other patients. In a companion paper, 515 patients with pathological stage I NSCLC were analysed. The tumours were classified in purely alveolar growth pattern (*n*=80, 16%) and angiogenic growth pattern (the remainder). The prognostic importance of the alveolar growth pattern was assessed. In the whole group, purely alveolar growth pattern did not have a prognostic value, but when the analyses were performed for the 137 patients classified as pT1N0, purely alveolar growth pattern (*n*=21) was associated with a poorer outcome (Pastorino *et al*, 1997). A subsequent study of the phenotypical characteristics of endothelial cells indicated that the alveolar growth pattern is nonangiogenic as the incorporated blood vessels do not express the integrin *α*V*β*3, a putative marker of endothelial cells participating in angiogenesis, and stain positively for LH39, expressed on mature basement membrane (Passalidou *et al*, 2002).

Our proposed classification is a modification of the classification by Pezzella *et al* and is based on the properties of the tumour–lung interface only. We believe that this is the region where the tumour expands and where the tumour–stroma interaction is most active. The highest mean microvessel density (MVD) and the highest VEGF/KDR (vascular endothelial growth factor bound to its receptor on endothelial cells)-activated microvessel density (aMVD) have been shown to be at the invading front of the tumours in NSCLC (Giatromanolaki *et al*, 2000; Koukourakis *et al*, 2000). Furthermore, the expression of other angiogenic factors, such as Angiopoietin I and Angiopoietin II have been principally observed at the edge of the tumour in NSCLC (Tanaka *et al*, 2002).

Our method differs from that of Pezzella *et al* by the use of H&E sections only for the classification into growth patterns without additional sections stained immunohistochemically with vascular markers. Our use of H&E sections has the advantage of allowing the classification into growth patterns to be carried out in routine daily pathological practice. It is possible that minimal angiogenesis might occur in broadened co-opted alveolar walls: in the minority of cases where this is a possibility, angiogenesis could be assessed using vascular immunostaining.

Our proposed system not only contains elements of a classification according to angiogenic profile but in addition also emphasises differences in the formation and destruction of stroma (Figure 1).

Noguchi *et al* (1995) proposed a new histological classification for small adenocarcinomas of the lung measuring 2 cm or less in greatest diameter. In this classification, the type A and B – that is, localised BAC without and with foci of collapse of alveolar structures – were associated with an excellent prognosis (100% 5-year survival). The type A and B are considered to be *in situ* peripheral adenocarcinomas. The growth pattern classification described in the present paper does not apply to *in situ* peripheral lung adenocarcinomas.

Other groups have studied the prognostic value of the alveolar growth pattern: Offersen *et al* (2001) studied 143 patients with different stages of NSCLC. The tumours were divided into two groups; alveolar (entirely or partially) and angiogenic. In a Cox multiple regression analysis for overall survival, advanced stage, higher age, adenocarcinoma and angiogenic vascular pattern were independent predictors of poor prognosis, while a better prognosis was found for the alveolar growth pattern. No representative tissue was available to examine the interface of the tumours in 46 patients (32%). The paraffin block containing the tumour tissue was chosen blindly in each patient, so no analysis of other sections was performed. Moreover, the median time to death for 112 patients was 22 months (range: 0–110), suggesting that operative mortality was not excluded. Finally, the papillary growth pattern (which was found by us to have an equally poor prognosis as the alveolar growth pattern and was exclusively present in adenocarcinoma) was classified by them in the angiogenic group. Our finding that the papillary growth pattern has a poor prognosis, similar to that of alveolar growth pattern, is a novel finding and has not been shown before.

In a recent study by Funai *et al* (2003), 204 surgically resected squamous cell lung carcinomas were analysed. A subset of 109 peripheral carcinomas were classified into three subtypes: (1) five cases of the alveolar space-filling type, (2) 54 cases of the expanding type and (3) 50 cases of the combined type. None of the five cases of the alveolar space-filling type showed any lymphatic vessel invasion, lymph node metastasis or pulmonary metastasis. The Kaplan–Meier survival estimate at 5 years for the alveolar space-filling type was 100%, as no event (death was the endpoint) was recorded for these patients. The authors concluded that a tumour of the alveolar space-filling type had the most favourable prognosis, might be an incipient carcinoma corresponding to squamous cell carcinoma *in situ*, and limited resection could be acceptable in this tumour type. These conclusions must be interpreted cautiously as the analyses were performed in a small subset of patients and results were based on univariate analysis of five patients.

The conclusion that patients with an alveolar, nonangiogenic growth pattern have a worse prognosis seems to be paradoxical in view of numerous studies describing an association between high MVD and poor prognosis. The rationale of counting microvessels in vascular ‘hot spots’ is that these areas originated from tumour cell clones with the highest angiogenic potential and, consequently, with easiest access to the blood stream and with an increased probability of producing metastases capable of becoming angiogenic and growing tumours (Vermeulen *et al*, 2002a). However, angiogenesis induces a chaotic and inefficient vascularisation. The vascularisation of the nonangiogenic growth pattern is more efficient, implying a better growth and progression for nonangiogenic tumour tissue. The prognostic value of MVD has been a controversial subject in NSCLC (Giatromanolaki *et al*, 1996; Chandrachud *et al*, 1997; Fontanini *et al*, 1997; Pastorino *et al*, 1997; Duarte *et al*, 1998; Matsuyama *et al*, 1998; Ohta *et al*, 1999; O'Byrne *et al*, 2000; Offersen *et al*, 2001; Tsoli *et al*, 2002). The largest study of 515 patients with the longest follow-up time found no prognostic value for MVD (Pastorino *et al*, 1997). In our view, this is because all studies contained tumours with different growth patterns, different angiogenic profiles and different prognosis (Sardari Nia *et al*, 2003). The prognostic value of MVD can only be investigated in the subgroup with an angiogenic growth pattern. Such a study has yet to be performed.

The clinical implications of our results are three-fold. Firstly, tumours with a nonangiogenic growth pattern will probably not respond to treatment with angiogenesis inhibitors. This resistance to antiangiogenic therapy will probably occur irrespective of whether a tumour is entirely or partially (only at the interface) nonangiogenic: nonangiogenic clones might be selected by antiangiogenic treatment. The growth pattern can be used as a surrogate marker of angiogenesis, and pretreatment patient selection based on the growth pattern could enlarge the fraction of patients responding to angiogenesis inhibitors.

Secondly, the proposed classification has a strong independent prognostic value for overall and disease-free survival. The growth patterns are a possible explanation for differences in survival of patients in the same stage. In stage I resected NSCLC, many patients are long-term survivors, but some relapse early and die. The patients with early relapse and reduced overall survival are those with an alveolar and papillary growth pattern. These ‘at risk’ patients may be identified based on the growth pattern and can be intensively followed or treated with adjuvant therapy.

Finally, in this study, we propose the hypothesis that the growth pattern of a tumour is the synthesis of different biological characteristics. The differences in clinical outcome and response to a specific treatment modality of patients in the same stage of disease are due to differences in biological characteristics of the tumours. This implies that different growth patterns might respond differently to specific treatment modalities, such as chemotherapy, antiangiogenic therapy and radiotherapy. This could provide new opportunities for the treatment of lung cancer and warrants further study.

In conclusion, the growth patterns identified have prognostic value additional to that provided by staging of the disease, and give a possible explanation for survival differences at the same stage. A prospective study would validate the results presented here and demonstrate the clinical usefulness of growth pattern identification.
